# Policy awareness among community-dwelling older adults: The roles of cognitive status and digital access

**DOI:** 10.1371/journal.pone.0357745

**Published:** 2026-10-15

**Authors:** Eun Kyung Jung, Hee Young Kim

**Affiliations:** 1 Department of Paramedicine, Wonkwang University, Iksan, Republic of Korea; 2 Department of Occupational Therapy, Honam University, Gwangju, Republic of Korea; Public Library of Science, UNITED STATES OF AMERICA

## Abstract

Policy awareness is a key prerequisite for the effective use of government programs among older adults. In increasingly digitalized information environments, access to policy information may depend not only on digital resources but also on cognitive capacity. However, limited evidence exists regarding whether the relationships between informational resources and policy awareness differ according to cognitive function. This study conducted a secondary analysis using data from the 2023 National Survey of Older Koreans, a nationally representative survey based on stratified multistage cluster sampling. The analytic sample included 9,951 community-dwelling adults aged 65 years or older. Policy awareness was measured using 11 items assessing awareness of government programs for older adults (range 0–22). Cognitive status was categorized using K-MMSE-2 scores into normal cognition, mild cognitive impairment, and moderate-to-severe cognitive impairment. Complex samples general linear models were applied to examine factors associated with policy awareness and to test interaction effects between cognitive status and key predictors. The weighted mean policy awareness score was 8.34 (SE = 0.13). Policy awareness differed significantly across cognitive groups, with the lowest scores observed among those with moderate-to-severe cognitive impairment and the highest among those with normal cognition. The overall model was statistically significant (Wald F = 14.59, p < 0.001) and the model explained 12.9% of the variance in policy awareness (R^2^ = 0.129). Significant interaction effects were observed between cognitive status and digital access (p < 0.001) and between cognitive status and depression (p = 0.003). The association between digital access and policy awareness appeared stronger among older adults with mild cognitive impairment compared with those with normal cognition. These findings suggest that cognitive status moderates the relationship between digital access and policy awareness. Improving digital access may be associated with higher levels of policy awareness among cognitively vulnerable older adults.

## Introduction

As populations age globally, a wide range of public policies have been developed to support the health and social participation of older adults. However, the mere existence of policies does not necessarily ensure that individuals can benefit from them. Awareness of policy information is a fundamental prerequisite for policy accessibility. In the context of aging policy, public policies are often implemented through specific government programs and services designed to support older adults. Therefore, in the present study, policy awareness refers to awareness of government-supported programs and services that operationalize broader public policy goals. Access involves the entire process through which individuals recognize needed services, seek and reach them, and ultimately obtain and use them [1]. In this context, policy awareness represents the initial stage through which individuals recognize relevant programs and translate that understanding into actual service use.

Previous studies have shown that higher levels of policy awareness are associated with greater health knowledge and more appropriate use of public services [2]. From this perspective, policy awareness can be viewed as a gateway through which individuals recognize and interpret policy information relevant to their needs.

In recent years, the environment for policy information delivery has rapidly shifted toward digital media. Digital platforms have increased the efficiency of information dissemination but have also introduced new challenges related to information overload. Excessive exposure to information may reduce information-processing capacity, lower decision-making quality, and increase cognitive burden [3,4]. The ability to utilize digital information is closely related to cognitive processes such as attention, working memory, and executive function [5]. Consequently, disparities in cognitive capacity may lead to inequalities in the ability to access and understand policy information. Recent studies have further highlighted that digital health literacy and multidimensional forms of digital exclusion—encompassing technological access, skills, and motivational factors—play an important role in shaping how older adults engage with digital information environments [6,7].

Existing studies have largely explained differences in policy awareness or service utilization among older adults in terms of sociodemographic factors such as age, education, income, social participation, and digital use [8]. According to digital divide theory, inequalities in access to and use of digital technologies may contribute to unequal access to information and public services. From a health literacy perspective, access to information alone does not guarantee the ability to understand, evaluate, and utilize that information effectively. However, recent research suggests that the digital divide is not solely a matter of technological access but also involves differences in information comprehension and usability. Digital interfaces often rely on text-based information, icons, and menu-based navigation systems that may impose cognitive demands during information search and interpretation [9–11]. Recent evidence suggests that higher levels of digital health literacy are associated with increased use of digital health resources and improved engagement with health-related information among older populations [12].

Cognitive function in later life should therefore be understood as a continuum rather than a binary distinction based solely on dementia diagnosis. Even mild levels of cognitive decline may influence information comprehension and decision-making processes, potentially affecting information-related outcomes such as policy awareness [13].

In addition to cognitive capacity, social and psychological factors may also influence engagement with policy information. Social interactions may serve as informal channels through which information about public programs spreads, potentially reducing information gaps among older adults with limited digital access [14,15]. Recent studies also suggest that digital engagement in later life is associated with improved psychological well-being and reduced depressive symptoms, partly through enhanced social connectivity and information access [16]. Furthermore, depressive symptoms have been associated with reduced motivation, lower levels of information-seeking behavior, and decreased social participation, which may limit exposure to policy-related information.

Despite these insights, limited research has examined whether the relationships between informational resources such as digital access and policy awareness differ according to cognitive function. Recent research on older adults’ social media use suggests that digital communication environments interact with eHealth literacy and information interpretation skills, highlighting the need to examine cognitive differences in information-related outcomes [17]. Understanding such differences is important for evaluating the equity and accessibility of policy delivery systems for older adults. Moreover, emerging evidence indicates that digital technologies in health and social care systems can improve access to services for older adults while simultaneously introducing new inequalities related to digital literacy and infrastructure [18].

Therefore, this study used nationally representative data from the 2023 National Survey of Older Koreans to examine factors associated with policy awareness among community-dwelling older adults. Specifically, this study investigated whether cognitive status moderates the relationships between digital access, depressive symptoms, social interaction, and policy awareness.

## Materials and methods

### Study design and participants

This study conducted a secondary analysis using data from the 2023 National Survey of Older Koreans conducted by the Korea Institute for Health and Social Affairs [19]. The survey is a nationally representative study of community-dwelling adults aged 65 years and older residing in 17 provinces of South Korea and uses a stratified multistage cluster sampling design.

The original survey included 10,078 participants. For the present study, respondents who completed the survey via proxy responses were excluded to ensure the validity of key variables including policy awareness, depression, and cognitive function. The final analytic sample consisted of 9,951 participants. When sampling weights were applied, the sample represented approximately 9,384,092 community-dwelling older adults in South Korea.

The original National Survey of Older Koreans received ethical approval from the Korea Institute for Health and Social Affairs. The present secondary analysis was reviewed by the Institutional Review Board of Wonkwang University and was granted exemption from ethical review (IRB No. 202512-106-01). This study was conducted using an anonymized dataset that was accessed for research purposes on 20 December 2025. The dataset did not contain any personally identifiable information, and the researchers did not have access to identifiable participant data at any time.

### Measures

#### Policy awareness.

Policy awareness was assessed using 11 items from the National Survey of Older Koreans that measured respondents’ awareness of government-supported programs and services for older adults [19]. These programs and services represent concrete policy instruments through which aging-related public policies are delivered to older adults. Each item was originally coded on a three-point scale (1 = “Do not know,” 2 = “Have heard of it but do not know it well,” 3 = “Know it well”). Responses were recoded as 0, 1, and 2 and summed to create a total score ranging from 0 to 22. Higher scores indicated greater awareness of government programs. Internal consistency in the present study was acceptable (Cronbach’s α = 0.809). The 11 programs included long-term care insurance, dementia-related services, elder protection services, and pension programs.

#### Cognitive function.

Cognitive function was assessed using the Korean version of the Mini-Mental State Examination-2 (K-MMSE-2) [13]. Scores range from 0 to 30, with higher scores indicating better cognitive functioning. Cognitive status was categorized into three groups: normal cognition (≥24), mild cognitive impairment (20–23), and moderate-to-severe cognitive impairment (≤19). The scale demonstrated acceptable reliability in this study (Cronbach’s α = 0.850).

#### Depression.

Depressive symptoms were measured using the Short Form of the Geriatric Depression Scale (SGDS-SF) [20]. The scale consists of 15 items with total scores ranging from 0 to 15. Higher scores indicate more severe depressive symptoms. The scale demonstrated good internal consistency in this study (Cronbach’s α = 0.850).

#### Digital access.

Digital access was assessed using 13 items reflecting engagement in digital activities in daily life, including sending and receiving messages, video calls, information searches, taking photographs or videos, listening to music, playing games, watching online videos, using social networking services (SNS), online shopping, online financial transactions, searching for and installing applications, and using kiosks [19]. Each item was coded dichotomously (0 = no, 1 = yes), and scores were summed to produce a total score ranging from 0 to 13. Higher scores indicated greater digital access. Internal consistency was high (Cronbach’s α = 0.891).

#### Social interaction.

Social interaction was measured using two variables assessing frequency of contact during the past year with (1) relatives or kin and (2) friends or neighbors [19]. Each variable was measured on a seven-point ordinal scale ranging from no contact to four or more contacts per week. For analysis, variables were reverse-coded so that higher values indicated more frequent contact.

#### Covariates.

Covariates included age, sex, educational attainment, household income quintile, marital status, household type, and number of chronic diseases**.**

### Statistical analysis

All analyses accounted for the complex sampling design of the survey, including stratification, clustering, and sampling weights. Descriptive statistics were calculated using weighted frequencies, percentages, means, and standard errors.

Group differences across cognitive function categories were examined using Rao–Scott χ² tests for categorical variables and complex samples t-tests for continuous variables.

Complex samples general linear models were conducted to identify factors associated with policy awareness. Independent variables included cognitive status, depressive symptoms, digital access, and social interaction variables. Interaction terms between cognitive status and the main predictors were included to examine moderating effects.

Continuous variables were mean-centered before creating interaction terms to reduce multicollinearity [21]. Regression coefficients are presented as unstandardized coefficients (B) with standard errors (SE) and 95% confidence intervals. Statistical significance was defined as p < 0.05. Multicollinearity was assessed using variance inflation factors. All analyses were conducted using IBM SPSS Statistics version 29.0 (IBM Corp., Armonk, NY, USA).

## Results

### Participant characteristics by cognitive function level

A weighted sample representing 9,384,092 community-dwelling older adults was included in the analysis. Among them, 12.4% were classified as having moderate-to-severe cognitive impairment, 19.6% had mild cognitive impairment, and 68.0% had normal cognitive function(Table 1).

**Table 1:** Weighted participant characteristics by cognitive function level (NSOK 2023).

Variable	Category / statistic	Moderate-to-severe	Mild	Normal	Total	p-value
**Sample distribution**	Weighted population size (POPSIZE, B estimate)	1234.97	1952.03	6768.11	9955.11	
	Weighted % of total	12.40%	19.60%	68.00%	100.00%	
Policy awareness score (0–22)	Mean (SE)	6.09 (0.25)	7.69 (0.20)	8.94 (0.14)	8.34 (0.13)	
**Covariates (control variables)**						
Age (years)	Mean (SE)	79.69 (0.46)	76.99 (0.27)	72.15 (0.14)	74.04 (0.14)	—
Sex	Male	27.30%	36.50%	49.30%	44.00%	<0.001
	Female	72.70%	63.50%	50.70%	56.00%	
Education level (recoded)	No formal education	46.60%	19.30%	3.40%	11.90%	<0.001
	Elementary school	28.20%	42.40%	24.10%	28.20%	
	Middle school	11.50%	20.80%	23.30%	21.40%	
	High school	13.30%	15.50%	39.40%	31.50%	
	College or higher	0.40%	2.30%	9.70%	7.00%	
Household income (quintiles)	Q1 (lowest)	32.00%	25.20%	16.30%	20.00%	<0.001
	Q2	28.10%	28.70%	15.90%	19.90%	
	Q3	17.70%	19.70%	20.40%	19.90%	
	Q4	13.00%	15.60%	22.80%	20.20%	
	Q5 (highest)	9.10%	10.80%	24.70%	20.00%	
Marital status (spouse)	With spouse	42.50%	50.40%	66.20%	60.10%	<0.001
	Without spouse	57.50%	49.60%	33.80%	39.90%	
Household type (4 categories)	Older adult living alone	46.50%	41.10%	28.10%	33.00%	<0.001
	Older adult couple	39.90%	47.40%	60.50%	55.30%	
	Living with adult children	13.00%	10.20%	9.40%	10.00%	
	Other	0.60%	1.40%	2.00%	1.70%	
Number of chronic diseases	Mean (SE)	2.80 (0.08)	2.53 (0.06)	1.93 (0.03)	2.15 (0.03)	—
**Main independent variables**						
Cognitive function (K-MMSE-2, 0–30)	Mean (SE)	14.83 (0.49)	21.76 (0.03)	27.23 (0.05)	24.62 (0.14)	—
Depression (SGDS-SF, 0–15)	Mean (SE)	5.00 (0.18)	3.89 (0.12)	2.55 (0.07)	3.12 (0.07)	—
Digital activity score (0–13)	Mean (SE)	1.90 (0.21)	3.06 (0.11)	5.60 (0.09)	4.64 (0.08)	—
Social contact: friends/neighbors (past year)	No contact	5.70%	1.70%	1.30%	1.90%	<0.001
	<Monthly	10.60%	8.10%	7.00%	7.60%	
	Monthly	7.80%	8.80%	8.80%	8.70%	
	2–3/month	15.30%	17.70%	17.60%	17.30%	
	Weekly	22.40%	22.60%	24.40%	23.90%	
	2–3/week	25.30%	27.70%	29.80%	28.90%	
	≥4/week	13.10%	13.30%	11.10%	11.80%	
Social contact: relatives/kin (past year)	No contact	28.20%	17.80%	11.20%	14.60%	<0.001
	<Monthly	42.10%	41.70%	39.50%	40.30%	
	Monthly	12.80%	16.20%	18.60%	17.40%	
	2–3/month	11.30%	16.30%	18.10%	16.90%	
	Weekly	3.30%	5.10%	7.70%	6.70%	
	2–3/week	1.80%	2.30%	3.80%	3.20%	
	≥4/week	0.40%	0.70%	1.10%	0.90%	

The weighted mean policy awareness score (range: 0–22) for the total sample was 8.34 (SE = 0.13). Policy awareness differed across cognitive function groups, with the lowest score observed among those with moderate-to-severe cognitive impairment (mean = 6.09, SE = 0.25), followed by those with mild cognitive impairment (mean = 7.69, SE = 0.20), and the highest score among cognitively normal older adults (mean = 8.94, SE = 0.14).

Significant differences in sociodemographic characteristics were observed across cognitive function groups (all p < 0.001). Participants with poorer cognitive function were older, more likely to be female, and more likely to have lower levels of educational attainment and household income. They were also less likely to live with a spouse and more likely to live alone.

Health-related characteristics also differed across cognitive groups. The mean number of chronic diseases was highest among participants with moderate-to-severe cognitive impairment (2.80 ± 0.08) and lowest among cognitively normal older adults (1.93 ± 0.03). Depressive symptoms were also more pronounced among participants with poorer cognitive function (5.00 ± 0.18) compared with those with normal cognition (2.55 ± 0.07).

Regarding key independent variables, digital activity scores increased with cognitive function level, with means of 1.90 (SE = 0.21) for the moderate-to-severe group, 3.06 (SE = 0.11) for the mild group, and 5.60 (SE = 0.09) for the normal group. Similarly, the frequency of social contact with relatives or kin and with friends or neighbors was higher among participants with better cognitive function.

### Interaction effects by cognitive function level

Complex samples general linear models were conducted to examine factors associated with policy awareness and to test interaction effects between cognitive function and key predictors(Table 2). The overall model was statistically significant (Wald F = 14.59, df1 = 29, df2 = 943, p < 0.001) and explained 12.9% of the variance in policy awareness.

**Table 2:** Complex Samples General Linear Model (CSGLM): tests of model effects for policy awareness.

Source	df1	df2	Wald F	p-value
Model (overall)	29	943	14.591	<0.001
Intercept	1	971	7.507	0.006
Cognitive status group	2	970	5.018	0.007
Depression (centered)	1	971	24.374	<0.001
Digital access (centered)	1	971	52.733	<0.001
Kin contact (centered)	1	971	5.488	0.019
Friend contact (centered)	1	971	13.270	<0.001
Age	1	971	21.217	<0.001
Number of chronic diseases	1	971	6.780	0.009
Sex	1	971	0.446	0.504
Education level	4	968	6.983	<0.001
Household income	4	968	0.523	0.719
Marital status	1	971	0.001	0.981
Household type	3	969	0.485	0.693
Cognitive status × Depression	2	970	5.705	0.003
Cognitive status × Digital access	2	970	7.906	<0.001
Cognitive status × Kin contact	2	970	0.544	0.581
Cognitive status × Friend contact	2	970	1.336	0.263

Note. Wald F tests account for the complex sampling design of the survey. p-values are two-sided.

Significant interaction effects were observed between cognitive function level and depressive symptoms (Wald F = 5.70, df1 = 2, df2 = 970, p = 0.003) as well as between cognitive function level and digital access (Wald F = 7.91, df1 = 2, df2 = 970, p < 0.001). In contrast, interaction terms between cognitive function and social contact variables were not statistically significant.

### Main effects of predictors of policy awareness

After accounting for interaction effects, several variables were significantly associated with policy awareness (Table 3). Digital access (Wald F = 52.73, p < 0.001), contact with relatives or kin (Wald F = 5.49, p = 0.019), contact with friends or neighbors (Wald F = 13.27, p < 0.001), age (Wald F = 21.22, p < 0.001), educational attainment (Wald F = 6.98, p < 0.001), and number of chronic diseases (Wald F = 6.78, p = 0.009) showed significant associations with policy awareness.

**Table 3:** CSGLM parameter estimates for policy awareness.

Predictor	B	SE	95% CI	t	p-value
Intercept	4.870	1.298	2.324, 7.416	3.753	<0.001
Cognitive status: Group 1 vs ref	−1.483	0.483	−2.431, −0.536	−3.072	0.002
Cognitive status: Group 2 vs ref	−0.288	0.182	−0.645, 0.068	−1.587	0.113
Depression (centered)	0.055	0.031	−0.006, 0.116	1.759	0.079
Digital access (centered)	0.235	0.031	0.175, 0.296	7.612	<0.001
Kin contact (centered)	0.196	0.063	0.072, 0.320	3.107	0.002
Friend contact (centered)	0.301	0.064	0.175, 0.426	4.700	<0.001
Age	0.073	0.016	0.042, 0.105	4.606	<0.001
Number of chronic diseases	−0.132	0.051	−0.232, −0.033	−2.604	0.009
Sex: category 1 vs ref	0.071	0.106	−0.138, 0.280	0.668	0.504
Education level: 1.00 vs ref	−1.710	0.371	−2.438, −0.982	−4.608	<0.001
Education level: 2.00 vs ref	−0.843	0.318	−1.466, −0.219	−2.651	0.008
Education level: 3.00 vs ref	−0.410	0.295	−0.990, 0.170	−1.388	0.165
Education level: 4.00 vs ref	−0.466	0.265	−0.987, 0.054	−1.760	0.079
Household income: 1.00 vs ref	−0.089	0.313	−0.704, 0.526	−0.283	0.777
Household income: 2.00 vs ref	−0.093	0.270	−0.622, 0.437	−0.343	0.731
Household income: 3.00 vs ref	0.068	0.235	−0.393, 0.529	0.290	0.772
Household income: 4.00 vs ref	0.168	0.190	−0.204, 0.540	0.885	0.376
Marital status: 1.00 vs ref	0.008	0.333	−0.645, 0.661	0.024	0.981
Household type: 1.00 vs ref	−0.558	0.582	−1.700, 0.583	−0.960	0.337
Household type: 2.00 vs ref	−0.503	0.511	−1.505, 0.499	−0.984	0.325
Household type: 3.00 vs ref	−0.340	0.531	−1.381, 0.701	−0.640	0.522
Cognitive status: Group 1 vs ref * Depression (centered)	0.183	0.055	0.076, 0.290	3.360	<0.001
Cognitive status: Group 2 vs ref * Depression (centered)	0.067	0.043	−0.017, 0.151	1.570	0.117
Cognitive status: Group 1 vs ref * Digital access (centered)	0.168	0.126	−0.079, 0.415	1.333	0.183
Cognitive status: Group 2 vs ref * Digital access (centered)	0.196	0.050	0.098, 0.294	3.930	<0.001
Cognitive status: Group 1 vs ref * Kin contact (centered)	−0.110	0.140	−0.385, 0.166	−0.781	0.435
Cognitive status: Group 2 vs ref * Kin contact (centered)	0.065	0.134	−0.198, 0.329	0.486	0.627
Cognitive status: Group 1 vs ref * Friend contact (centered)	−0.076	0.108	−0.288, 0.137	−0.700	0.484
Cognitive status: Group 2 vs ref * Friend contact (centered)	−0.163	0.102	−0.362, 0.037	−1.597	0.111

Note. Centered covariates: dep_c, dig_c, kin_c, frd_c.

Regression coefficient estimates indicated that digital access (B = 0.235, p < 0.001), contact with relatives or kin (B = 0.196, p = 0.002), and contact with friends or neighbors (B = 0.301, p < 0.001) were positively associated with policy awareness. Age was also positively associated with policy awareness (B = 0.073, p < 0.001), whereas the number of chronic diseases showed a negative association (B = −0.132, p = 0.009). Sex, household income, marital status, and household type were not significantly associated with policy awareness.

### Visualization of the interaction between cognitive function and digital access

To illustrate the interaction effect, predicted policy awareness scores were calculated based on the regression coefficients from the complex samples general linear model. Continuous covariates were held at their sample means, and categorical variables were set to their reference categories. Policy awareness increased as digital access increased across all cognitive groups (Fig 1, Table 4).

**Table 4:** Adjusted Means (Estimated Marginal Means) by cognitive status group.

Cognitive status	Adjusted mean	SE	95% CI
Moderate to severe cognitive impairment	7.690	0.538	6.634–8.746
Mild cognitive impairment	8.940	0.230	8.489–9.391
Normal cognition	9.134	0.188	8.765–9.502

**Note.** Estimated marginal means were obtained from the complex samples general linear model (CSGLM). Continuous covariates were held at their sample means, and categorical covariates were fixed at reference categories. Digital access (dig_c) was set at its mean value when calculating adjusted means.

**Fig 1:**
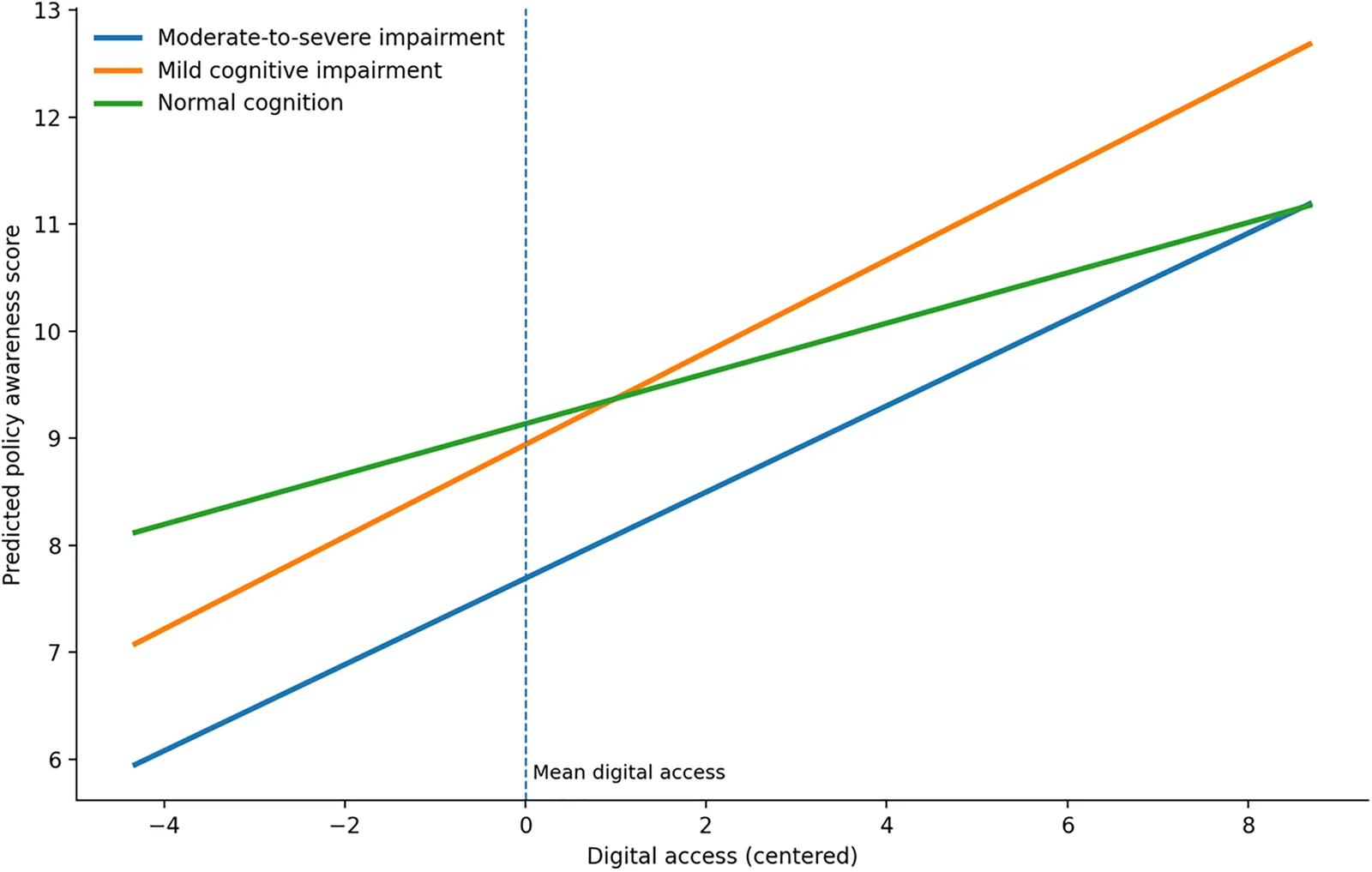
Interaction between cognitive status and digital access in predicting policy awareness. Predicted policy awareness scores according to levels of digital access across three cognitive status groups.

However, the slope of this relationship differed across cognitive function levels. The interaction analysis indicated that the relationship between digital access and policy awareness differed across cognitive function groups. In particular, the positive association between digital access and policy awareness appeared stronger among individuals with mild cognitive impairment than among those with normal cognition. Estimated marginal means calculated at the mean level of digital access showed that policy awareness remained highest among older adults with normal cognition, followed by those with mild cognitive impairment and those with moderate-to-severe cognitive impairment. Furthermore, the steeper slope observed among participants with mild cognitive impairment suggests that increases in digital access were associated with relatively greater gains in policy awareness compared with cognitively normal older adults.

## Discussion

This study examined factors associated with policy awareness among community-dwelling older adults and investigated whether cognitive function moderates the relationships between digital access, depressive symptoms, social interaction, and policy awareness. The findings indicate that cognitive function plays an important role in shaping how informational resources are associated with policy awareness.

First, older adults with poorer cognitive function demonstrated lower levels of policy awareness. This finding is consistent with previous studies showing that cognitive decline affects information processing, comprehension, and decision-making abilities [22,23]. Cognitive capacity is closely linked to health literacy and the ability to understand complex information structures, which may influence awareness of government programs and services.

Poorer cognitive function may affect not only opportunities to access information but also the ability to retain, interpret, and act upon information once it is received. Older adults with cognitive impairment may have difficulty remembering policy information or understanding administrative procedures, which may contribute to lower levels of policy awareness. This interpretation is consistent with previous evidence suggesting that cognitive decline influences not only information access but also information comprehension and decision-making processes.

Second, the association between digital access and policy awareness varied depending on cognitive status. Although digital access was positively associated with policy awareness overall, the relationship appeared stronger among individuals with mild cognitive impairment compared with those with normal cognition. This pattern may reflect differences in baseline levels of digital engagement. Older adults with lower cognitive function may have fewer opportunities to access digital information environments, and therefore increases in digital access may lead to relatively larger improvements in policy awareness.

Previous research has suggested that digital technologies can both expand access to information and create new inequalities related to digital literacy and information-processing capacity [24]. Digital platforms often require users to navigate complex information structures, evaluate multiple sources, and interpret administrative content. These processes rely on cognitive resources such as attention and working memory, which may vary across individuals.

In addition to digital access, social interaction was positively associated with policy awareness. Contact with relatives, friends, and neighbors may provide informal channels through which information about government programs and services spreads. Prior studies have highlighted the importance of social networks in facilitating access to health information and public resources among older adults [11,12].

Interestingly, the interaction between social contact variables and cognitive function was not statistically significant. This finding suggests that social interactions may function as relatively accessible information channels regardless of cognitive status. Unlike digital platforms, interpersonal communication may impose lower cognitive demands and may therefore remain an effective pathway for information dissemination among cognitively vulnerable populations.

Functional status may also influence policy awareness. Individuals with limitations in activities of daily living (ADL) or instrumental activities of daily living (IADL) may encounter barriers to obtaining, understanding, and acting on policy-related information. Because functional limitations are closely related to cognitive decline and may partly lie on the pathway linking cognitive status and policy awareness, these variables were not included in the present model. Future studies should examine whether functional status mediates or moderates the relationship between cognition and policy awareness.

Although previous studies have reported gender differences in digital engagement, health literacy, and use of public services, sex was not significantly associated with policy awareness after adjustment for other covariates in the present study. This finding suggests that educational attainment, cognitive status, and digital access may account for much of the variation previously attributed to gender differences among older adults.

Although the explanatory power of the model was modest (R² = 0.129), policy awareness is a multidimensional construct that is likely influenced by factors not captured in the present dataset, including health literacy, trust in government institutions, prior experiences with public services, and individual information-seeking preferences. Furthermore, awareness of government programs and services does not necessarily translate into actual utilization, suggesting that additional barriers may exist between information acquisition and service use.

The findings suggest that improving digital access among older adults may help reduce disparities in policy awareness. However, expanding digital access alone may not be sufficient if cognitive barriers to information comprehension remain unaddressed. Policy communication strategies should therefore consider the cognitive heterogeneity of older populations.

Approaches such as simplifying policy information, incorporating visual materials, and combining digital platforms with community-based outreach programs may help improve accessibility for cognitively vulnerable groups. Integrating digital and non-digital communication channels may be particularly important for ensuring equitable policy access among older adults.

Several limitations should be considered when interpreting the findings of this study. First, the cross-sectional design precludes causal inferences regarding the relationships among cognitive status, digital access, and policy awareness. Second, policy awareness was measured as self-reported familiarity with government programs and services and should not be interpreted as actual access to or utilization of those services. Third, although the study adjusted for a range of sociodemographic and health-related factors, other potentially relevant variables, such as health literacy, trust in government institutions, and prior experiences with public services, were not available in the dataset. Finally, functional status, including activities of daily living (ADL) and instrumental activities of daily living (IADL), was not included in the present analysis. Future studies should further examine the role of functional abilities in shaping policy awareness among older adults.

## Conclusion

This study examined factors associated with policy awareness among community-dwelling older adults and investigated whether cognitive function moderates the relationship between digital access and policy awareness. The results indicate that digital access is positively associated with policy awareness and that this association varies across cognitive function levels.

Although older adults with higher cognitive function demonstrated higher overall levels of policy awareness, increases in digital access were associated with relatively larger gains in policy awareness among those with mild cognitive impairment. These findings highlight the importance of considering cognitive differences when designing policy communication strategies for older populations.
